# The COVID-19 Infection Resulted Delayed Esophagogastroduodenoscopy in Patients Admitted with Variceal Bleeding: Hospital-Based Outcomes of a National Database

**DOI:** 10.3390/diseases11020075

**Published:** 2023-05-21

**Authors:** Mustafa Gandhi, Zahid Ijaz Tarar, Umer Farooq, Saad Saleem, Harleen Kaur Chela, Ebubekir Daglilar

**Affiliations:** 1 Department of Medicine, University of Missouri School of Medicine, Columbia, MO 65211 USA; mustafahgandhi@gmail.com (M.G.);; 2 Rochester General Hospital, Rochester, NY 14621, USA; 3 Department of Medicine, Sunrise Hospital and Medical Center, Las Vegas, NV 89109, USA; 4 Department of Gastroenterology and Hepatology, Charleston Area Medical Center, West Virginia University School of Medicine, Charleston, WV 25304, USA

**Keywords:** hospital outcomes, COVID-19 disease, upper gastrointestinal bleeding, endoscopy

## Abstract

During the COVID-19 pandemic in 2020, most healthcare services, including inpatient and outpatient procedures, got delayed. We reviewed the effect of COVID-19 infection on the timing of esophagogastroduodenoscopy (EGD) in variceal bleeding patients and analyzed the complications of delayed EGD. Using the National Inpatient Sample (NIS) 2020, we identified patients admitted for variceal bleeding with COVID-19 infection. We performed a multivariable regression analysis and adjusted it for patient and hospital-related variables. The International Classification of Disease Tenth Revision (ICD-10) codes were used for patient selection. We measured the effect of COVID-19 on the timing of EGD and further analyzed the effect of delayed EGD on hospital-based outcomes. A total of 49,675 patients diagnosed with variceal upper gastrointestinal bleeding were analyzed, out of which 915 (1.84%) were COVID-19 positive. Variceal bleeding patients who were COVID-positive had a significantly lower rate of EGD performed within the first 24 h of admission (36.1% vs. 60.6% *p* = 0.001) compared to the patients who tested negative for COVID-19. The performance of EGD within 24 h of admission resulted in a decrease in all-cause mortality by 70% (adjusted odds ratio (AOR) 0.30, 95% CI 0.12–0.76, *p* = 0.01) compared to EGD after 24 h. A significant decrease was noted in the odds of ICU admission rate (AOR 0.37, 95% CI 0.14–0.97, *p* = 0.04) in patients who got EGD within the first 24 h of admission. No difference in odds of sepsis (AOR 0.44, 95% CI 0.15–1.30, *p* = 0.14) and vasopressor use (AOR 0.34, 95% CI 0.04–2.87, *p* = 0.32) was seen in COVID positive vs. COVID negative group. The hospital mean length of stay (2.14 days, 95% CI 4.35–0.06, *p* = 0.06), mean total charges ($51,936, 95% CI $106,688–$2816, *p* = 0.06), and total cost (11,489$, 95% CI 30,380$–7402$, *p* = 0.23) was similar in both COVID-positive and -negative groups. In our study, we found that the presence of COVID-19 infection in variceal bleeding patients resulted in a significant delay in EGD compared to COVID-negative patients. This delay in EGD resulted in increased all-cause mortality and intensive care unit admissions.

## 1. Introduction

COVID-19 is an infectious disease caused by the SARS-CoV-2 virus, which first emerged in China and then rapidly spread around the world. Although initial reports suggested the zoonotic spread of the infection through food markets in Wuhan, later, it was recognized that human-to-human droplet spread plays a major role in transmission. According to the World Health Organization (WHO), COVID-19 has affected about 517 million people and resulted in around 15 million deaths, either directly from COVID-19 infection or secondary to the burden on the healthcare system due to the coronavirus pandemic, which leads to delayed patient care [1].

COVID-19 most commonly affects the respiratory system with a clinical syndrome ranging from asymptomatic infection to a severe, rapidly progressive fatal disease. It is associated with a variety of systemic and respiratory manifestations such as cough, fever, dyspnea, pulmonary edema, pneumonia, acute respiratory distress syndrome (ARDS), and systemic inflammatory response syndrome progressing to multiorgan dysfunction [2]. Even after recovery from the illness, debilitating fibrosis occurs in the lungs. Similar to other coronaviruses, COVID-19 infection also leads to significant extra-pulmonary disease, which affects other organ systems, such as the gastrointestinal (GI) and cardiovascular systems. Although certain symptoms are caused by the direct invasion and toxicity of the cells to the virus, other manifestations are attributed to the exaggerated immune response of the host cells to the viral invasion. The most common gastrointestinal symptoms are nausea, vomiting, and diarrhea. Studies have also reported a 2–13% incidence of gastrointestinal bleeding and conditions such as acute liver injury, acute cholecystitis, acute pancreatitis, and mesenteric ischemia in hospitalized COVID-19 patients [2,3]. In a study of the gastrointestinal complications in critically ill patients with severe COVID-19 infection, Kaafarani et al. discovered that the common gastrointestinal complications were transaminitis, varying degrees of ileus, including colonic paralytic ileus, bowel ischemia secondary to vascular thrombosis and gastrointestinal bleeding [3].

A cohort study of 11,158 analyzing the prevalence and risk factors for gastrointestinal bleeding in hospitalized patients with COVID-19 found that the point prevalence of GI bleed was about 3% (314 patients). There was no higher risk for GI bleeding in patients on anti-coagulation or anti-platelet agents, and no other significant risk factors were identified. However, patients that develop gastrointestinal hemorrhage during hospitalization did have a high risk of mortality [4]. Another retrospective study by Prasoppokakorn et al. analyzed the risk factors of upper GI bleeding in patients with COVID-19 infection and discovered that the only significant factors associated with a higher risk of active upper GI bleeding were the absence of proton pump-inhibitor (PPI) therapy and a high Glasgow- Blatchford score [5]. Prophylaxis with PPI was found to lower the incidence of upper and lower gastrointestinal bleeding. The severity of COVID-19 infection, anticoagulants, antiplatelets, or steroid use was not associated with any significant difference in the groups with and without gastrointestinal bleeding [5]. The postulated mechanisms of increased gastrointestinal bleeding are hemorrhagic colitis, bowel wall inflammation, corticosteroid use, stress ulcers, or variceal bleeding [4,5].

## 2. Background

Cirrhosis is progressive scarring and fibrosis of the liver that is characterized by morphological changes in the liver, which can be associated with hepatic dysfunction. The most common causes of cirrhosis in the developed world include chronic viral hepatitis (hepatitis B and C), non-alcoholic fatty liver disease, alcohol-related liver disease, and hemochromatosis. Less common causes include primary and secondary biliary cirrhosis, autoimmune hepatitis, medication-induced liver injury, Wilson’s disease, and primary sclerosing cholangitis. Once the signs and complications of hepatic dysfunction set in, it is termed decompensated cirrhosis as opposed to compensated cirrhosis which is the absence of hepatic dysfunction and complications. The major complications of cirrhosis are ascites, variceal bleeding, hepatorenal syndrome, hepatic encephalopathy, spontaneous bacterial peritonitis, hepatocellular carcinoma, and hepatopulmonary syndrome.

Variceal bleeding is the second most common complication in this patient group which typically presents as hematochezia or melena. In addition, the esophageal varices more commonly lead to variceal bleeding compared to gastric varices, with endoscopic band ligation recommended as the standard of care [6]. 

The general measures to control gastrointestinal hemorrhage include [6]:

- A restrictive blood transfusion strategy to maintain a hemoglobin level of more than 7 mg/dl;
- Antibiotics prophylaxis with intravenous ceftriaxone for a minimum of 7 days, as bacterial infections are a major cause of mortality in patients with variceal bleeding;
- Proton pump inhibitor therapy is more beneficial in improving outcomes of ulcer-related GI bleeding compared to variceal bleeding;
- Reversal of coagulopathy has shown conflicting evidence of benefit;

Specific therapy for when esophageal varices are the cause of hemorrhage [6]:

- Intravenous splanchnic vasoconstrictors such as octreotide, terlipressin, or somatostatin have been shown to improve outcomes when initiated early and continued for 2 to 5 days;
- Endoscopic therapy, once hemodynamic stability has been attained, is the standard of care treatment, with early EGD being preferred over late EGD;
- Trans jugular intrahepatic portosystemic shunt is a viable treatment option for patients who have failed other forms of therapy.

There are increased rates of complications and mortality in COVID-19 patients with pre-existing decompensated liver cirrhosis when compared to those without liver cirrhosis [7]. This is possibly due to the immunocompromised state in decompensated cirrhotic patients in addition to coagulopathy and hepatic synthetic dysfunction. Furthermore, some reports have suggested that COVID-19 may contribute to variceal hemorrhage in patients with liver cirrhosis [7]. Prior studies reported not only a higher incidence of GI bleeding in COVID-19 patients as but worse outcomes and increased mortality as well [4,5].

During the COVID-19 pandemic, the gastroenterology service, such as other hospital services, had been adversely affected by delays and logistical constraints for inpatient procedures. In addition, there was an increased burden on the gastroenterology service due to gastrointestinal manifestations such as diarrhea, nausea, vomiting, abdominal pain, and gastrointestinal hemorrhage in patients with COVID-19 infection, along with laboratory findings of elevated liver enzymes and hyperbilirubinemia. In addition to the endoscopy on a COVID-19 patient being a higher-risk procedure, the endoscopy staff is at elevated risk of contracting an infection from procedure-related aerosol generation and virus shedding in stool, which also leads to decreases in elective endoscopic procedures [8]. 

Although there are reported data on the increased incidence of gastrointestinal bleeding, worse hospital outcomes, and predictors of bleeding in COVID-19 patients but data on the timing of endoscopy in this population and the impact of endoscopy timing on hospital-based outcomes is scarce. In our analysis, we reviewed the effect of EGD timing on the patients admitted for variceal bleeding in the presence of COVID-19 infection. 

## 3. Study Design and Database Description

It is a retrospective study of adult patients hospitalized due to variceal bleeding performed on the Nationwide Inpatient Sample (NIS) 2020 database. It is designed as a stratified probability sample representative of all non-federal acute care hospitals nationwide. A 20% probability sample from all hospitals is then collected. Each hospital discharge is then weighted (weight = total number of discharges divided by the number of discharges included in the 20% sample), which makes it nationally representative. The dataset for 2020 consists of more than 6 million discharges (*n* = 6,471,165). This data represents a 20% stratified sample from 4580 non-federal acute care hospitals in 49 states. This estimates to be about 32 million yearly discharges nationwide when weighted and is representative of 95% of hospital discharges nationwide [9]. 

## 4. Study Patients

Patients with International Classification of Diseases, Tenth Revision, and Clinical Modification (ICD-10-CM) codes for VUGIB were included in this study. Patients with and without COVID-19 were separated among both patient groups. Patients were excluded if they were younger than eighteen years of age. The outcomes were compared based on the performance of early EGD (defined to be completed within 24 h of admission). 

## 5. Statistical Analysis

Analyses were performed using STATA, version MP 17 (StataCorp, College Station, TX, USA). We used univariable logistic regression to compute unadjusted odds ratios (ORs). Subsequently, multivariable regression models were built by including all confounders significantly associated with the outcome on univariable analysis with a cutoff *p*-value of 0.2 or less. Variables deemed clinically crucial to the outcome based on the literature review were included in the model irrespective of whether they were significantly associated with the outcome univariable analysis. The potential confounders that were adjusted for were: age in years, gender, race, admission day as the weekend or weekday, median income in the patient’s zip code as four hierarchical categories, patient co-morbidities as measured by the Deyo adaptation of the Charlson Co-morbidity Index for administrative data, hospital location, hospital region, hospital teaching status as teaching or non-teaching, and hospital bed size. We also added major co-morbidities included in the Rockall score in the regression model (including heart failure, ischemic heart disease, renal failure, liver failure, metastatic cancer, and the presence of shock) to control for confounding by indication. As laboratory values were not available in the database, MELD-Na and Child-Pugh Class could not be used; however, we developed a qualitative surrogate severity marker based on some of the variables in MELD-Na and Child-Pugh Class (presence of ascites, international normalized ratio (INR) abnormalities, hepatic encephalopathy, hyponatremia, and hypoalbuminemia) to adjust for liver disease severity in our analysis additionally [10]. 

## 6. Results

**Figure 1:**
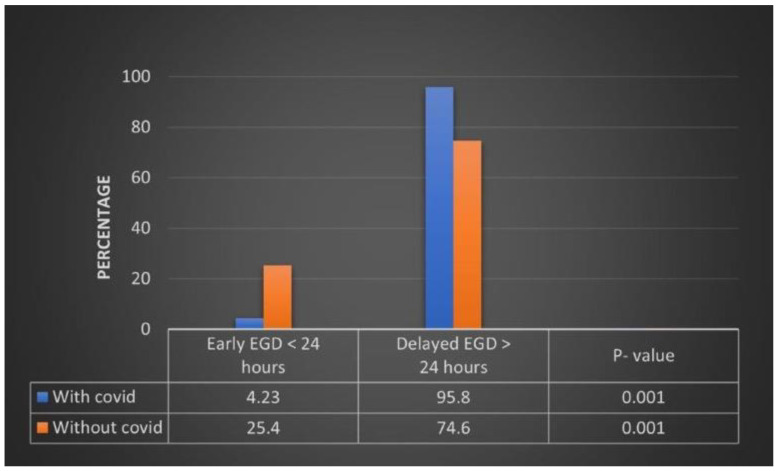
Percentage of early vs. delayed EGD in patients with and without COVID-19.

- Baseline characteristics of Variceal hemorrhage patients: Forty-nine thousand six hundred seventy-five patients were admitted with a principal diagnosis of variceal bleeding, of which 915 (1.84%) have co-existent COVID-19. On analysis of baseline characteristics of these patients based on the timing of EGD, we found that the mean age of the patients in the early EGD group was 55.4 compared to 56.4 in the delayed (more than 24 h) EGD group. Females comprised 28.8% of the study population in the early EGD group compared to 28.2% in the delayed EGD group. Most of the patients in the early and delayed EGD group were white (58.7% vs. 43.5%, *p* = 0.08), followed by Hispanics (25.4% vs. 39.1, *p* = 0.05), black (4.76% vs. 8.7%, *p* = 0.31), native Americans (6.35% vs. 3.48%, *p* = 0.40), Asians (3.17% vs. 0.09%, *p* = 0.27), and others (1.595 vs. 4.35%, *p* = 0.34) respectively. A more significant percentage of patients in the early EGD group fell in a higher Charlson co-morbidity index group (89.4% vs. 88.9%, *p* = 0.005). The early or delayed EGD group saw no significant difference in median household income. The rate of early and delayed EGD was similar in different hospitals based on the regional distribution, hospital size, and teaching versus non-teaching status. Examining distribution by insurance status shows us that patients in the early and delayed EGD group have similar insurance. Table 1 details the baseline characteristics of the study population. (Table 1)
- Percentage of Early and Delayed EGD in Variceal bleeding patients with COVID-19: Among 915 patients with COVID-19 disease, 330 (36.1%) received EGD within the first 24 h, whereas 29,549 (60.6%) out of 48,760 non-COVID-patients-underwent EGD within the first 24 h. This result achieved statistical significance (*p* = 0.001). (Figure 1)
- Adjusted odds of Outcomes based on the timing of EGD in the variceal bleeding group: On multivariate regression analysis, we found that performing EGD < 24 h resulted in a significant decrease in all-cause mortality by 70% (AOR 0.30, 95% CI 0.12–0.76, *p* < 0.001) compared to delayed EGD > 24 h after admission. Performing EGD early also decreases the rate of intensive care unit admission (AOR 0.37, 95% CI 0.14–0.97, *p* = 0.04). No difference in the odds of sepsis (AOR 0.44, 95% CI 0.15–1.29, *p* < 0.14), Blood transfusion (AOR 1.64, 95% CI 0.79–3.40, *p* = 0.18), and vasopressor use (AOR 0.39, 95% CI 0.05–2.79, *p* = 0.35) based on the timing of the EGD. Performing EGD earlier did not result in a shorter mean length of hospital stay (−2.14 days, 95% CI −4.35–0.06, *p* = 0.06), mean hospital charges (−$23,647, 95% CI −83,388–36,094, *p* = 0.44), and total cost (−$11,489, 95% CI −30,380–7402, *p* = 0.23) (Table 2).
- Quarterly rate of EGD in COVID-19 patients with variceal bleed in 2020: We divided 2020 into three quarters and analyzed the rate of early vs. late EGD and found that 70% of variceal bleed patients with co-existent COVID infection in 1st quarter received EGD after 24 h and only 30% underwent EGD within the first 24 h of presentation. In the second (May–Aug) and third quarter (Sep–Dec), 36.9% and 37.2% of the variceal bleed patients with covid infection received EGD within 24 h of presentation. No statistical difference was found in the rate of EGD throughout the year (Table 3).

## 7. Discussion

Upper endoscopy after initial resuscitation is the gold standard therapy for upper gastrointestinal bleeding (UGIB) [11]. The timing goal per some guidelines is to be able to perform an endoscopy within 12 h of the presentation of variceal bleeding, while other societies recommend that upper endoscopy be performed within 24 h in patients with an acute variceal UGIB [12,13]. For the purposes of our study analysis, we have used the 24 h mark to classify whether an EGD was performed early (less than 24 h after the presentation of bleeding) or late (more than 24 h after the presentation of bleeding). An upper endoscopy serves the dual purpose of diagnostics as well as therapeutics and can be performed by all trained gastroenterologists. Prior to performing an EGD, erythromycin can be administered to clear the stomach of blood contents by acting as a prokinetic agent. Before we delve into discussing the implications of our study results, we shall briefly review the modes of therapy at our disposal with an EGD to control an acute variceal bleed.

- Endoscopic variceal ligation (EVL): This is the most commonly performed therapeutic procedure for variceal bleeding and is the preferred initial treatment. It involves the placement of elastic bands on the culprit bleeding vessels within the distal 5 cm of the esophagus;
- Endoscopic sclerotherapy (ES): When EVL fails, sclerotherapy is the next modality used by endoscopists to control variceal bleeding. It involves the injection of a sclerosant agent such as ethanolamine or sodium morrhuate into the bleeding vessel and brings about thrombosis of the varices;
- Esophageal stenting: There have been recent promising trials of the use of a specially designed self-expanding metal stent (SEMS) for the therapy of refractory acute esophageal variceal bleeding. The SEMS is expanded under visual guidance over the endoscope without the use of fluoroscopy.

Endoscopic variceal ligation and sclerotherapy are very effective, with various studies reporting success rates of 70 to 100%. Although most of the trials performed are older, both EVL and ES have been shown to have similar efficacy when it comes to the control of bleeding. However, EVL is superior to ES when outcomes of rebleeding, stricture formation, and death are compared. Variceal band ligation and sclerotherapy can be used for the treatment of both esophageal varices and gastroesophageal varices. Gastroesophageal varices can be either along the lesser curvature (Type I) or along, the greater curvature of the stomach (Type II). However, it is important to note that isolated gastric varices cannot be treated by these techniques.

Numerous studies have demonstrated that band ligation has a better safety profile when compared to endoscopic sclerotherapy. The lower complication rate of EVL is hypothesized to be due to the lesser depth of tissue damage. In addition, a major advantage of endoscopic band ligation is the much lower rate of stricture formation. Some of the complications from endoscopic sclerotherapy include ulceration, portal hypertensive gastropathy, esophageal perforation, and mediastinitis. 

Although there is data on gastrointestinal bleeding in COVID-19 patients, the literature on EGD-related outcomes in these patients is limited. On analysis of variceal upper gastrointestinal bleeding (VUGIB) outcomes based on the timing of EGD, we discovered that patients who underwent EGD sooner had better outcomes compared to those who underwent EGD later (more than 24 h after admission). Although early upper endoscopy in VUGIB patients led to a decrease in all-cause mortality (about 70%) and intensive care unit admission rates, there was no significant difference in length of hospital stay, hospital costs, sepsis, or blood transfusions. The rate of ICU admission with early EGD was likely a result of prompt diagnosis and control of the bleed, thereby preventing clinical decline. 

Garg et al. performed a National Inpatient Sample 2007–2013 analysis to determine if early EGD improved mortality outcomes, length of hospital stay, and costs [14]. Their results showed that patients with late EGD had 50% higher mortality odds than those who underwent early EGD. There was a three times higher likelihood of death in patients who did not receive EGD than those who had early EGD [14]. EGD within 24 h was associated with lower mortality, morbidity, hospital cost, and length of stay. A notable point that differentiates this study from ours is that Garg et al. included all causes of upper gastrointestinal bleeding, of which esophageal variceal bleeding formed 12% of the total sample. Additionally, none of the patients had co-existing COVID-19 infection.

A retrospective cohort study was performed by Guo et al. in Hong Kong, which analyzed the outcomes in patients with acute upper gastrointestinal bleeding from 2013 to 2019 based on the timing of the endoscopy [15]. About 12.7% of the total cases of bleeding were associated with varices after adjustment of baseline characteristics. None of the patients had documented COVID-19 infection. Patients were categorized into three groups based on endoscopy timing: urgent (within 6 h of presentation), early (6 to 24 h of presentation), and late (more than 24 h after presentation). The results showed that those who underwent EGD from 6 to 24 h had lower mortality and ICU admission rates compared to the other groups. One important finding of this study was that outcomes of variceal bleeding were worse only in the late endoscopy group (more than 24 h after admission which concurs with our study results. It also suggests that the mortality and outcomes of variceal bleeding may be dependent on other prognostic factors, such as the severity of liver disease and other co-morbidities. Our study shows that patients with COVID-19 infection were more likely to undergo delayed EGD compared to non-COVID patients and have worse outcomes. These worse outcomes can be attributed to both the delay in EGD as well as the COVID infection, which is a significant co-morbidity (acts as a prognostic factor to determine outcomes in variceal bleeding).

On similar lines, a randomized controlled trial by Lau et al. analyzed the 30-day mortality in patients with acute UGIB with a Glasgow-Blatchford score of greater than 12 who underwent urgent (within 6 h) versus early (6 to 24 h) EGD [11]. Again, 44 out of the 516 patients had a variceal cause of bleeding. Again, there were no significant differences in 30-day mortality or rebleeding between these groups.

Jung D.H. et al. conducted a meta-analysis in 2020 to study the effect of performing early (<12 h) versus late (>12 h) EGD for acute variceal bleeding [16]. They discovered no significant differences in the overall mortality and rebleeding rates, thus enforcing the importance of appropriate timing of EGD over urgent EGD tailored to an individual patient’s clinical condition. This contrasts with our study, where we found significant improvements in all-cause mortality and ICU admission rates in early EGD because our study used the 24-h mark to differentiate between early vs. late. In contrast, Jung D.H. et al. used the 12-h mark. This suggests that 24 h is a more appropriate EGD timeline to aim for in this patient group. 

The results of most of the studies align with our findings, generally demonstrating reduced mortality, morbidity, and cost to the healthcare system. It is noteworthy that none of these prior studies were performed on COVID patients, but their findings are comparable to our results. Despite the demonstrated advantages of early EGD, only 36.1% of patients with COVID and variceal UGIB underwent an EGD within 24 h, highlighting the need for systems to ensure sooner endoscopic evaluation and treatment in these patients. Based on the outcomes of variceal bleeding based on EGD timings from prior studies and our analysis, the most appropriate time seems to be from about 6 h to 24 h after the presentation. This allows ample time for resuscitation with intravenous fluids, blood products, proton pump inhibitor therapy, and splanchnic vasoconstrictors to ensure a more stable clinical condition of the patient while going for the endoscopy. A 24 h time frame is also early enough that the fatal progression of the bleed is limited. 

The proposed causes of delay in EGD during the COVID pandemic are most likely due to logistic considerations, with equipment, funds, and personnel involved in tackling the COVID-19 pandemic. There was a severe strain and shortage of healthcare resources, with personnel, equipment, and funds being diverted to treating COVID-19 pneumonia and the containment of the pandemic in general. A high burden of burnout, infection, stress, emotional strain, and financial hardships was endured by healthcare workers during this period [17]. Our study highlights how the timing of the EGD in COVID patients with UGIB affects patients and the healthcare system, which could act as a catalyst to introduce interventions to prevent delay in endoscopy. This could improve outcomes and reduce healthcare costs.

Our study’s strengths lie in the diverse patient population based on the National Inpatient Sample database, which includes all acute care hospitals from 49 states. This indicates the strong power of the study nationwide and generalizability. Given that there is scarce data on outcomes of variceal UGIB in COVID patients based on the EGD timing, our study sheds light on these parameters. Our study presents the most updated data on the effect of delayed EGD on hospital-based outcomes of variceal bleeding patients. We used the latest NIS data in which ICD-10 coding is used, which is more specific and reliable.

A possible shortcoming of this study is the inability to account for the quality of supportive resuscitation measures and COVID-19 treatment in patients presenting with upper gastrointestinal bleeding that could affect outcomes. There is also no data on the severity of COVID-19 illness in the sample population, which could independently affect the outcomes. Furthermore, this is an observational study, so randomization was not possible. Finally, NIS is an administrative database and is susceptible to coding bias [18]. 

## 8. Conclusions

Esophagogastroduodenoscopy is the standard of care for the treatment of acute variceal gastrointestinal bleeding. Most guidelines recommend early rather than late EGD in these cases due to better outcomes. Our analysis of the outcomes of early vs. late EGD in COVID-19 patients with acute VUGIB revealed better comes in those who underwent an EGD within 24 h of presentation compared to those who underwent EGD after 24 h. Early EGD was associated with lower all-cause mortality and intensive care unit admission. Additionally, patients with COVID-19 were more likely to undergo EGD later compared to those who were not infected with COVID-19. This disparity can be explained by the logistic, financial, and personnel constraints that the gastroenterology services were burdened by during the pandemic.

## Figures and Tables

**Table 1:** Baseline characteristics of the study population.

Baseline Characteristics	Variceal Hemorrhage Early EGD < 24 h	Variceal Hemorrhage Delayed EGD > 24 h	p Value
Mean Age [years]	55.4	56.4	0.61
Women [n (%)]	95 (28.8%)	165 (28.2%)	0.93
Race [n (%)]			
White	185 (58.7%)	250 (43.5%)	0.08
Black	15 (4.76%)	50 (8.7%)	0.31
Hispanic	80 (25.4%)	225 (39.1%)	0.05
Asians	10 (3.17%)	5 (0.09%)	0.27
Native Americans	20 (6.35%)	20 (3.48%)	0.40
Others	5 (1.59%)	25 (4.35%)	0.34
Charlson Co-Morbidity Index [n (%)]			
0	0	0	0
1	15 (4.55%)	45 (7.69%)	0.42
2	20 (6.06%)	20 (3.42%)	0.40
3 or more	295 (89.4%)	520 (88.9%)	0.005
Median Household Income in Zip Code (Quartile) *			
1st (0–25th)	110 (33.9%)	235 (40.9%)	0.34
2nd (26th–50th)	75 (23.1%)	115 (20%)	0.62
3rd (51st–75th)	65 (20%)	130 (22.6%)	0.68
4th (76th–100th)	75 (23.1%)	95 (16.5%)	0.35
Hospital Region [n (%)]			
Northeast	65 (19.7%)	85 (14.5%)	0.35
Midwest	90 (27.3%)	105 (18.0%)	0.17
South	100 (30.3%)	230 (39.3%)	0.23
West	75 (22.7%)	165 (28.2%)	0.46
Insurance Status [n (%)]			
Medicare	110 (33.3%)	195 (33.3%)	1.0
Medicaid	90 (27.3%)	215 (36.7%)	0.21
Private/Self-pay	95 (28.8%)	90 (15.4%)	0.03
Uninsured	20 (6.06%)	65 (11.1%)	0.24
Hospital bed size [n (%)]			
Small	80 (24.2%)	120 (20.5%)	0.55
Medium	95 (28.8%)	195 (33.3%)	0.53
Large	155 (47.0%)	270 (46.2%)	0.92
Hospital teaching status [n (%)]			
Rural	10 (5.52%)	10 (7.30%)	0.56
Urban non-teaching	50 (19.6%)	70 (17.5%)	0.55
Urban teaching	270 (74.9%)	505 (75.2%)	0.44

* Median household income for the patient’s Zip Code: 1st Quartile: $1–49,999$, 2nd quartile: $50,000–$64,999. 3rd quartile: $65,000–$85,999, 4th quartile: $86,000+.

**Table 2:** Adjusted odds of outcomes based on the timing of endoscopy in variceal bleeding patients.

Outcomes	Adjusted Odds Ratio	95% CI	*p*-Value
All-cause mortality	0.30	0.12–0.76	<0.001
Sepsis	0.44	0.15–1.29	0.14
Intensive care admission	0.37	0.14–0.97	0.04
Blood transfusion	1.64	0.79–3.40	0.18
Vasopressor use	0.39	0.05–2.79	0.35
Mean length of stay (days)	−2.14	−4.35–0.06	0.06
Total charges	−23,647$	−83,388$–36,094$	0.44
Total cost	−11,489	−30,380–7402	0.23

**Table 3:** Quarterly rate of EGD in COVID-19 patients with variceal bleed in 2020.

Quarters	Early EGD% <24 h	Delayed EGD% >24 h
1st Quarter (January–April 2020)	30	70
2nd quarter (May–August 2020)	36.9	63.1
3rd Quarter (September–December 2020)	37.2	62.8

*p*-value: 0.84.

## Data Availability

For data supporting research results please reach out directly to the authors.
